# Mortality of oncohematologic patients admitted to the intensive care unit (icu) who required invasive mechanical ventilation (imv). do we have to delay intubation?

**DOI:** 10.1186/2197-425X-3-S1-A246

**Published:** 2015-10-01

**Authors:** Y Corcia Palomo, T Knight Asorey, I Espigado Tocino, L Martín Villén, A Gutiérrez Pizarrayo, J Garnacho Montero

**Affiliations:** Intensive Care Service, H Virgen del Rocio, Sevilla, Spain; Haematology and Haemotherapy Service, H Virgen del Rocio, Sevilla, Spain; Red Española de Investigación en Patología Infecciosa REIPI, H Virgen del Rocio, Sevilla, Spain

## Intr

The oncohematologic patients (OHP) with respiratory insufficiency who are admitted in the ICU have high mortality, even more if they need support with invasive mechanical ventilation (IMV). Respiratory support with non-invasive ventilation (NIV) tries to avoid intubation and therefore reduce mortality, although this is not yet fully proven.

## Objectives

Study mortality difference between OHP admitted to the ICU with respiratory failure who were intubated during the first hours and those who were intubated after more than 24 hours of support with NIV.

## Patients and Methods

We include all OHP admitted to our ICU with respiratory failure during two years (2013 and 2014). Demographic characteristics, underlying disease, admission cause, presence of organ failure, severity scores and mortality were registered. Data were compared by analysis bivariate between those intubated within 24 hours (group 1) and those requiring VMI after more than 24 hours of support with NIV (group 2).

## Results

49 patients, of whom 42 required VMI were included. 35 belonged to group 1, and 7 patients to group 2. We observed that group 1 patients have a significantly higher APACHE II [28 (22-34) vs 17 (16-23); p = 0.04] and more situations of septic shock [20 (57.1) vs. 1 (14.3); p = 0.005]. No significant differences in the development of organ failure (renal, hepatic, hematologic), the use of supportive therapies, or in-hospital mortality (30, 85.7% vs. 6, 85.7%; p = 1) were found.

## Conclusions

In our series, the failure of NIV carries the same mortality as the VMI in the early hours of ICU admission.

